# Supplementary material for: Development of Composite Sponge Scaffolds Based on Carrageenan (CRG) and Cerium Oxide Nanoparticles (CeO2 NPs) for Hemostatic Applications
Source: Biomimetics (Basel). 2023 Sep 4;8(5):409. doi: 10.3390/biomimetics8050409 (PMC10527261; doi:10.3390/biomimetics8050409)
Supplement: Supplementary file 1 [file biomimetics-08-00409-s001.zip › biomimetics-2559564-supplementary.pdf]

CRG **CRG-CeO_2-0.5** **CRG-CeO_2-1**

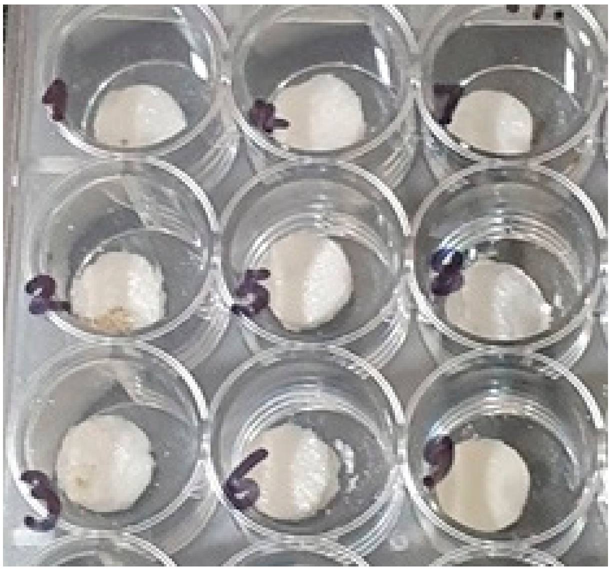

Figure S1. Photo of CRG, CRG-CeO_2-0.5, and CRG-CeO_2-1 scaffold after immersion in PBS

TEM provided by supplier SEM images of CeO_2 NPs

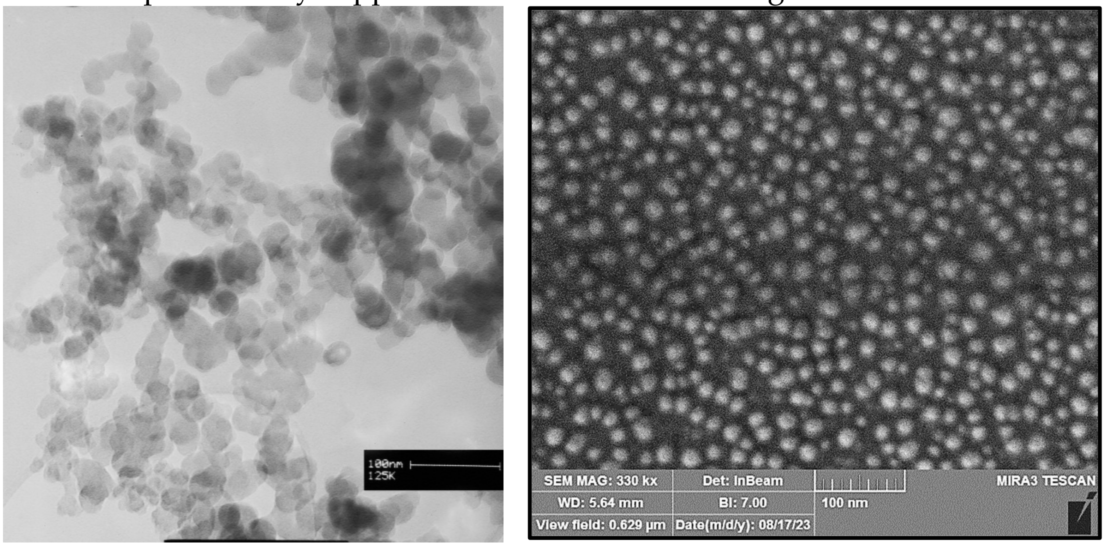

Figure S2. TEM and SEM Photo of CeO_2 NPs
